# Primary central nervous system lymphoma presenting as a pure third ventricular lesion: a case report

**DOI:** 10.1186/1752-1947-5-213

**Published:** 2011-05-28

**Authors:** Mehdi Sasani, Muzaffer Bayhan, Hadi Sasani, Tuncay Kaner, Tunc Oktenoglu, Gokhan Cakiroglu, Ali Fahir Ozer

**Affiliations:** 1 American Hospital Neurosurgery Department, Nisantasi, Istanbul, Turkey; 2 International Hospital Neurosurgery Department, Yesilkoy, Istanbul, Turkey; 3 Istanbul University Medical Faculty Radiology Department, Fatih, Istanbul, Turkey; 4 International Hospital Pathology Department, Yesilkoy, Istanbul, Turkey

## Abstract

**Introduction:**

Primary central nervous system lymphomas are infrequently occurring lymphomas that account for only 0.3-1.5% of all intra-cranial neoplasms in patients without acquired immune deficiency syndrome. However, a pure third ventricle lymphoma is extremely rare. Here, we discuss the similar radiological appearances of lesions localized in the third ventricle and the importance of accurately diagnosing primary central nervous system lymphomas for favorable treatment outcomes.

**Case presentation:**

A 38-year-old Caucasian man from Turkey presented with a severe headache lasting for three months that failed to respond to any medication. Both severity and duration of the symptoms increased gradually, resulting in vomiting, nausea and gait disturbance that accompanied the headache for three weeks. Neuro-imaging studies showed a lesion located solely in the third ventricle, resulting in partial obstruction of the foramen of Monro. The pre-operative diagnosis was a colloid cyst. Following the surgical procedure, the results of pathological and immunochemical assays revealed that the pre-operative diagnosis was incorrect and that the lesion was a primary central system lymphoma.

**Conclusion:**

Pure third ventricle lymphomas are extremely rare and are exceptionally localized. It is important to be aware of, and to differentiate between, other possible third ventricular lesions that may mimic the same radiological appearance. Accurate diagnosis is necessary for selecting appropriate treatment modalities.

## Introduction

Primary central nervous system lymphomas (PCNSL) are infrequently occurring lymphomas that account for only 0.3-1.5% of all intra-cranial neoplasms in patients without acquired immune deficiency syndrome (AIDS) [1,2]. Patients with AIDS, congenital immune deficiencies and those undergoing organ transplantations are at a greater risk of developing this condition. The most common localization sites of PCNSL (both B and T cells type) are in the supra-tentorial white matter of the frontal parietal lobes [3].

Here we report an unusual PCNSL involving only the third ventricle. Indeed, few cases have been reported in the literature. We will also discuss other more common third ventricle masses that may mimic the radiological characteristics of PCNSL.

## Case Presentation

**Figure 1:**
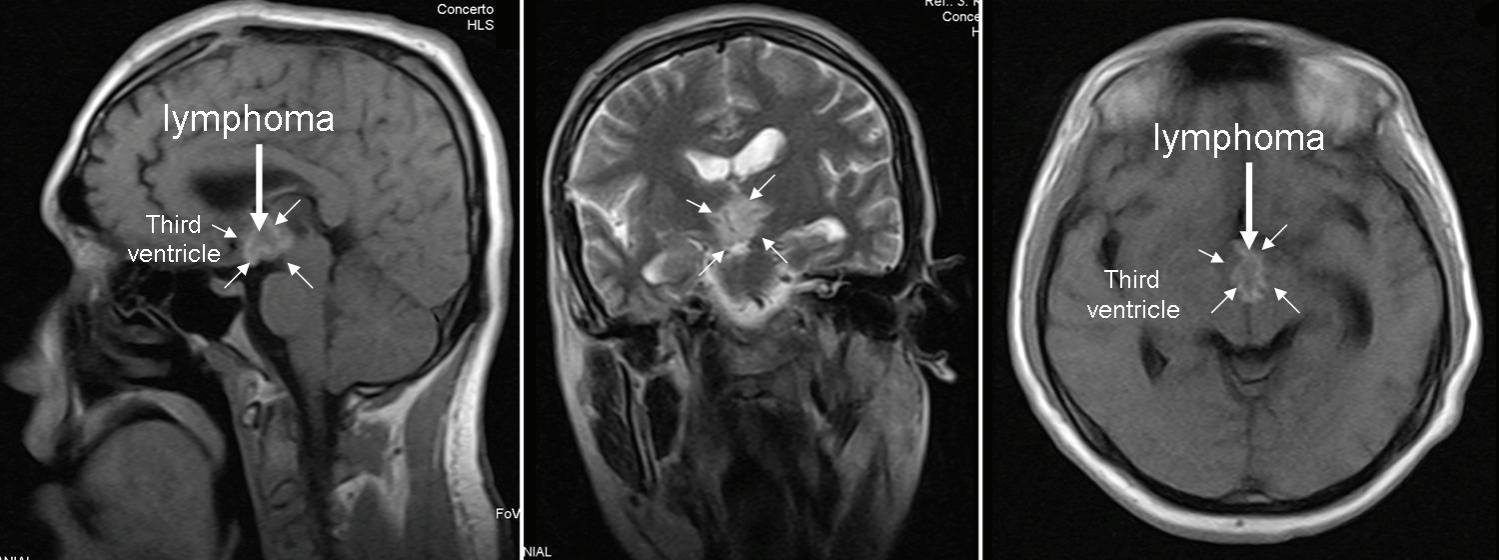
**A pre-operative cranial MRI showing the primary lymphoma (arrow), which was solely located in the third ventricle (small arrows)**.

A 38-year-old male (Caucasian) patient from Turkey presented with a persistent severe headache with a duration of approximately three months. Both the severity and duration of the symptoms increased gradually during this time. Vomiting, nausea, and temporary gait disturbance were present during the three weeks leading up to his hospital visit. According to his clinical history, his headache did not respond to any prescribed medication, and his complaints did not correspond to a specific posture. Hematological parameters were within normal limits, and the serological test for human immunodeficiency virus was negative. Visual field and visual quality were tested with standard clinical finger confrontation. The results of tests were free of abnormal findings. A fundoscopic examination revealed that his optic disc margins, ratio of artery to vein sizes and venous pulsation were within normal limits. Mid-line cerebellar function was tested by having our patient walk on a straight line, and hemispheric cerebellar function was tested by having our patient perform rapid alternating movements and by rapidly having him touch his nose or the physician's moving index finger. The examinations of cerebellar, ganglionic, and cortico-spinal pathways were all normal. The results of all of these neurological examinations were within normal limits and suggested that our patient did not have any symptoms of neurological disturbance. To identify the underlying pathology, magnetic resonance imaging (MRI) was performed. This examination revealed that the third ventricle was completely filled with a mass-like lesion that was hypointense on T1-weighted scans and hyperintense on T2-weighted scans. The lesion was partially obstructing the foramen of Monro; however, it did not cause severe hydrocephalus (Figure 1). Our patient underwent surgery for a craniotomy.

An intra-hemispheral transcallosal approach was performed, allowing access to his right ventricle and the foramen of Monro. Due to tumoral expansion, the foramen of Monro was dilated. Although analysis of a frozen section was consistent with PCNSL, the tumor was completely resected without complication.

**Figure 2:**
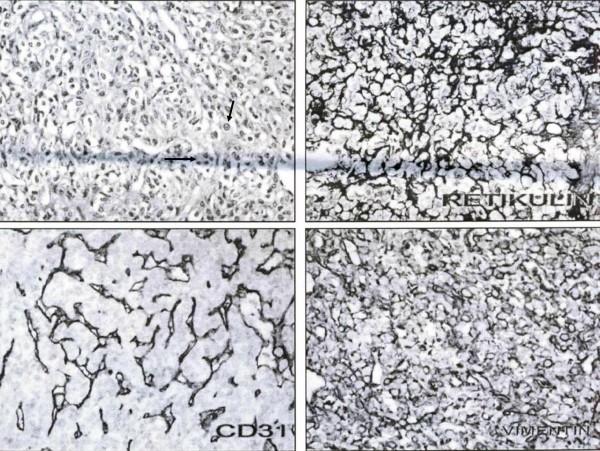
**Histopathological examination showed increased lymphoma sheets of round cells with small to moderate amounts of cytoplasm (black arrows)**.

Histopathological examination revealed increased lymphoma sheets of round cells with small to moderate amounts of cytoplasm (Figure 2). Our patient underwent a staging evaluation that included a whole-body positron emission tomography/computed tomography (PET/CT) scan, bone marrow biopsy and analysis of blood chemistry. All of these procedures failed to reveal any abnormalities. The PET/CT scan showed a slight accumulation of 2-[^18^F] fluoro-2-deoxyglucose in the original location of the tumor.

**Figure 3:**
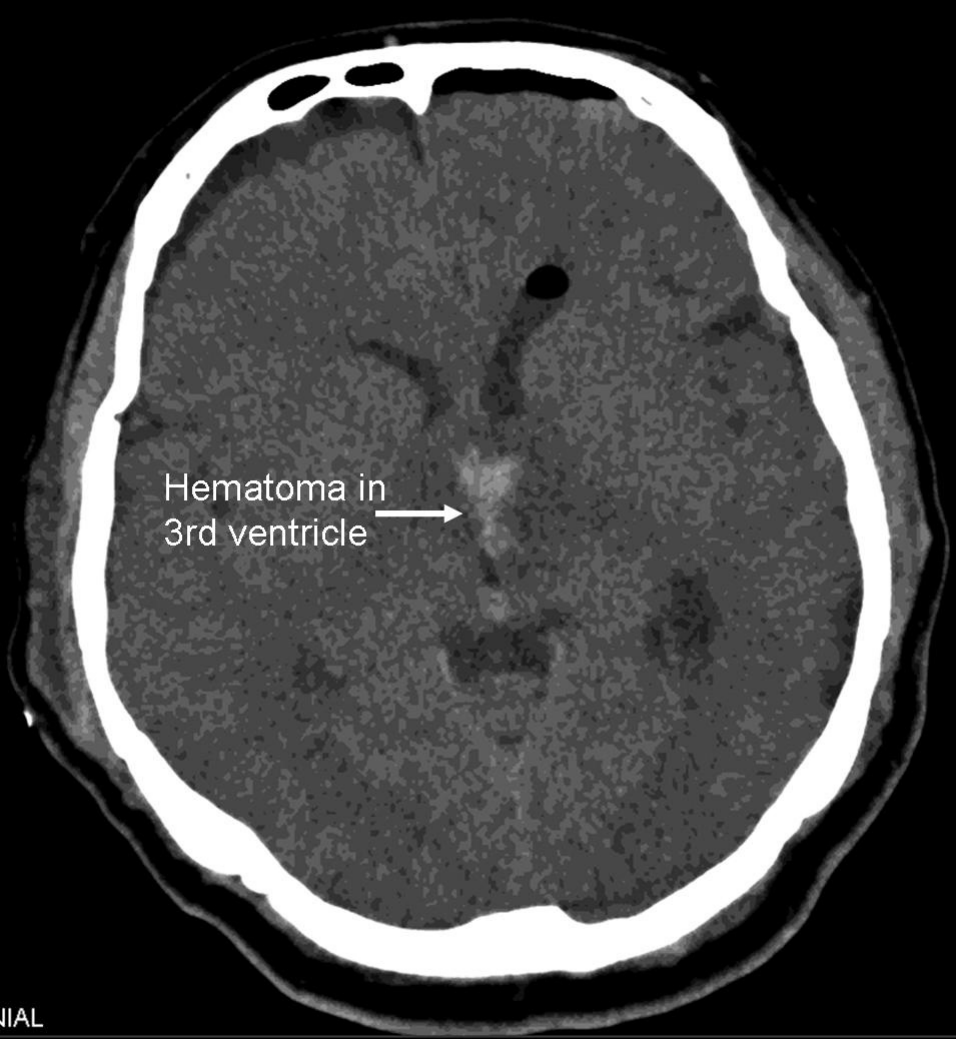
**A cranial CT scan performed three days post-operatively showing a hematoma in the surgery field with no indications of hydrocephalus**.

**Figure 4:**
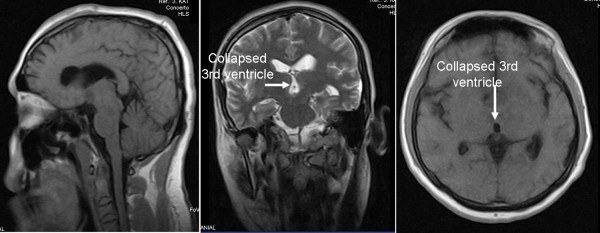
**A follow-up cranial MRI performed six months post-operatively showed no recurrence of lymphoma and collapsed third ventricle**.

A post-operative cranial computed tomography (CT) scan concluded soon after surgery revealed a hematoma in the third ventricle (Figure 3). The neurological examination was free of symptoms. Follow-up CT scans were conducted and revealed a blood clot that obstructed the foramen of Monro, resulting in hydrocephalus. However, the follow-up cranial CT scan did not show the hydrocephalus, which indicated that the hematoma was no longer present. Our patient refused all treatment options except corticosteroids. A follow-up cranial MRI conducted six months after surgery showed that the lymphoma had not reoccurred (Figure 4). Our patient was available for follow-up after one year.

## Discussion

PCNSLs are infrequent tumors that account for 0.7-0.9% of all lymphomas and only 0.3-1.5% of intra-cranial tumors [1]. These can occur in both immune-competent and immune-compromised patients [1]. Reports suggest that this type of lymphoma occurs more commonly in men than women, in a ratio of 3:2 (men:women) [1,4]. Intra-cranial lymphomas are diagnosed using both morphological criteria and immunohistochemical reactions [5]. Most primary intra-cranial lymphomas are comprised of non-Hodgkin's B-cells [6]. Cerebrospinal fluid analysis yields a cytological diagnosis in fewer than half of patients with B-cell PCNSL. Neuro-imaging modalities can also reveal solitary lesions, which are most commonly located supra-tentorially, in the white matter of the frontal or parietal lobes or in the sub-ependymal regions. However, lesions may also appear in the deep gray matter [3]. Typically, these lesions are in the central gray matter (33%), the basal ganglia-thalamus-hypothalamic region (17%), the cerebral white matter near the corpus callosum (55%), the posterior fossa (11%) and the peri-ventricular region. Fewer than 1% of cases reported occurred within the spinal cord [1,6,7]. The involvement of the third ventricle in PCNSL cases is quite rare and thus is considered to be exceptional. B-cell primary intra-cranial lymphoma typically presents in patients approximately 50 years of age and is more common in male patients [6]. The patient may present with a large variety of symptoms, such as an alteration in mental status, followed by nausea, headache, hemiparesis, alterations in cerebellar function, cranial nerve palsies and visual deterioration [6,8].

The findings from radiological imaging of the third ventricular lymphoma can easily be confused with other more common lesions that share the same localization [9], including a colloid cyst, cranio-pharyngioma, hypothalamic and thalamic glioma, ependymoma, basilar tip aneurysm and neuro-cytoma. An MRI is very useful for differentiating intra-cranial masses, particularly from cystic lesions such as a colloid cyst. Unfortunately the radiographic description of PCNSL is poor at best, especially given the sporadic and limited involvement of the cerebrospinal fluid and vitreous matter.

Ueda *et al. *[10] reported that all lesions showed hypointensity in MRI T1-weighted images, whereas three lesions showed definite hypointensity to gray matter and others showed hyperintensity in T2-weighted images. There was, however, no pathological difference between the hyperintensive and hypointensive lesions in the T2-weighted images. In addition, Gualdi *et al. *[11] demonstrated that neoplastic processes localized on the floor of the third ventricle are frequently responsible for neurological and dysendocrine symptoms. Furthermore, the results of this study suggest that CT and MRI studies are the most reliable neuro-imaging techniques for the diagnostic and surgical management of neoplastic masses affecting this region.

Treatment for intra-cranial lymphoma can include chemotherapy, radiotherapy (RT), surgery and a combination of these treatment modalities [12]. In the present case, our patient refused all treatments except corticosteroids. Corticosteroid treatment typically leads to a significant tumor regression that is often associated with clinical improvement [13]. The neuro-imaging response can be dramatic, sometimes showing complete remission of contrast-enhancing abnormalities. Most responses, however, are temporary, although complete remission has been reported [14].

In this case, the pre-operative diagnosis based on the findings of an initial MRI incorrectly indicated that the lesion was a colloid cyst. The accurate post-operative diagnosis, however, was PCNSL, which is rarely observed, particularly if it is a pure third ventricle lymphoma. It is essential to note that if the pre-operative diagnosis had been correct, unnecessary surgical procedures may have been avoided, and the patient's treatment might have been more appropriate (that is, steroid therapy, radiotherapy or chemotherapy). Indeed, PCNSL is sensitive to steroids (40% combined with RT) and is highly radiosensitive (80-90%) [15].

## Conclusion

Pure third ventricle lymphomas are extremely rare and do not normally occur with exceptional localization. Thus, for proper diagnosis, it is important to differentiate between other possible third ventricle lesions that may mimic the radiological appearance of such lymphomas. It is equally important to obtain accurate diagnostic results because the correct differentiation determines treatment options.

## Consent

Written informed consent was obtained from the patient for publication of this case report and any accompanying images. A copy of the written consent is available for review by the Editor-in-Chief of this journal.

## Competing interests

The authors declare that they have no competing interests.

## Authors' contributions

MS examined the patient, interpreted the findings and performed the surgery, and was a major contributor in writing the manuscript. MB examined the patient, interpreted the findings and performed the surgery. HS analyzed and interpreted the radiologic examination findings. TK designed and reviewed the manuscript. TO esigned and reviewed the manuscript. GC analyzed and interpreted the pathologic examination findings. AFO managed the authors' accordance and attended in surgery. All authors read and approved the final manuscript.
